# Global Genomic Epidemiology of *Escherichia coli* (ExPEC) ST38 Lineage Revealed a Virulome Associated with Human Infections

**DOI:** 10.3390/microorganisms10122482

**Published:** 2022-12-15

**Authors:** Erica L. Fonseca, Sergio M. Morgado, Raquel V. Caldart, Ana Carolina Vicente

**Affiliations:** 1 Laboratório de Genética Molecular de Microrganismos, Instituto Oswaldo Cruz—FIOCRUZ, Rio de Janeiro 21040-360, RJ, Brazil; 2 Centro de Ciências da Saúde, Universidade Federal de Roraima, Boa Vista 69300-000, RR, Brazil

**Keywords:** phylogenomics, zoonosis, high-risk, genomic epidemiology, high-pathogenicity island, accessory genome, ETT2

## Abstract

Background: Most of the extraintestinal human infections worldwide are caused by specific extraintestinal pathogenic *Escherichia coli* (ExPEC) lineages, which also present a zoonotic character. One of these lineages belongs to ST38, a high-risk globally disseminated ExPEC. To get insights on the aspects of the global ST38 epidemiology and evolution as a multidrug-resistant and pathogenic lineage concerning the three axes of the One Health concept (humans, animals, and natural environments), this study performed a global phylogenomic analysis on ST38 genomes. Methods: A phylogenetic reconstruction based on 376 ST38 genomes recovered from environments, humans, livestock, and wild and domestic animals in all continents throughout three decades was performed. The global information concerning the ST38 resistome and virulome was also approached by in silico analyses. Results: In general, the phylogenomic analyses corroborated the zoonotic character of the ExPEC ST38, since clonal strains were recovered from both animal and human sources distributed worldwide. Moreover, our findings revealed that, independent of host sources and geographic origin, the genomes were distributed in two major clades (Clades 1 and 2). However, the ST38 accessory genome was not strictly associated with clades and sub-clades, as found for the type 2 T3SS ETT2 that was evenly distributed throughout Clades 1 and 2. Of note was the presence of the *Yersinia pestis*-like high-pathogenicity island (HPI) exclusively in the major Clade 2, in which prevails most of the genomes from human origin recovered worldwide (2000 to 2020). Conclusions: This evidence corroborates the HPI association with successful *E. coli* ST38 establishment in human infections.

## 1. Introduction

*Escherichia coli* is a commensal bacterium and a versatile pathogen capable of causing intestinal and extraintestinal infections. Extraintestinal pathogenic *E. coli* (ExPEC) presents a complex phylogeny and comprises many lineages with enhanced metabolic repertoires that affect not only humans’ but also animals’ health, besides occurring in the environment [1,2,3,4,5]. Due to its remarkable genome plasticity, the ExPEC group is able to colonize a variety of host sites, and includes uropathogenic *E. coli* (UPEC), neonatal meningitis *E. coli* (NMEC), sepsis-associated *E. coli* (SEPEC), and avian pathogenic *E. coli* (APEC) [6].

The ST38, together with other ExPEC lineages (ST131, ST405, and ST648), emerged recently as the globally dominant strains isolated from extraintestinal infections. The treatment of infections caused by such ExPEC lineages has become a challenge to public health due to the increase in the prevalence of multidrug-resistant strains presenting resistance to the first-line and the last-resort antibiotics [7], raising concerns about how ExPEC, such as ST38, evolves and diversifies in terms of virulence and antibiotic resistance. 

Interestingly, some ST38 strains may present a uropathogenic/enteroaggregative hybrid character, due to the presence of both intestinal and extraintestinal virulence traits, which may contribute to the enhancement of ST38 virulence [8,9]. In spite of this, there are few studies focusing on the characterization of clinical hybrid strains [10]. 

Moreover, the ST38 has been associated with the global dissemination of the extended spectrum β-lactamase (ESBL)-coding *bla*_CTX-M_ gene [1,2,4]. On the other hand, studies on the occurrence of carbapenemase genes, such as *bla*_NDM_ and *bla*_OXA_, in ST38 are scarce. Particularly, a recent study on ST38 harboring the carbapenemase OXA-244 and several virulence genes relevant to the establishment of infections suggested the emergence of a supranational clonal outbreak, at least, in Europe [11]. 

Therefore, based on the recent concern about ST38 impact on the three axes of the One Health concept (humans, animals, and environment), mainly considering its role in spreading antibiotic resistance, and the gaps regarding genomic studies on ST38 lineage and its epidemiology in a global context, we performed a large-scale comparative genomic analysis on publicly available ST38 genomes in order to gain insights into the antibiotic resistance, virulence, adaptability, and colonization capabilities of this lineage. 

## 2. Materials and Methods

### 2.1. EnteroBase ST38 Meta-Analysis

EnteroBase is integrated software that supports the identification of global population structures within several bacterial genera [12]. Here, a meta-analysis based on the geographical information of 3290 ST38 entries included in EnteroBase (https://enterobase.warwick.ac.uk accessed on 29 November 2022) was performed (Table S1). The ST38 epidemiological map was generated using R software v4.1.1 with different libraries (maptools and ggplot2). 

### 2.2. Comparative Genomic Analyses and In Silico Characterization of the Resistome and Virulome

All complete and draft available *E. coli* genomes (*n* = 24,102) were retrieved from GenBank and EMBL databases. The seven MLST alleles that define the ST38 (*adk-4*, *fumC-26*, *gyrB-2*, *icd-25*, *mdh-5*, *purA-5*, and *recA-19*) were separately used as queries in BLASTn analyses to recover all currently available ST38 genomes. The completeness of the draft ST38 genomes was assessed by using the BUSCO v5.3.2 tool, presenting a minimum completeness >93% [13]. A total of 376 ST38 genomes (complete and draft) recovered on all continents from humans, wild and companion animals, livestock, vegetables, poultry, sewage, fresh water, and seawater throughout three decades (1983–2020) were used in comparative genomic analyses. The genome sequence of one *E. coli* strain (EC35) (GenBank assembly accession no. GCA_019492145) recovered in 2016 from a urinary tract infection in a clinical setting in the Amazonian city of Boa Vista, Roraima, Brazil, was obtained in this study and also included in the analyses. The genomic DNA extraction was obtained with NucleoSpin Microbial DNA kit (Macherey-Nagel), and the genome libraries were constructed using Nextera paired-end libraries. The sequencing was performed by Illumina Hiseq 2500, generating reads of 250 bp length. The raw reads were filtered and trimmed using the NGS QC Toolkit v.2.3.3 [14], with a Phred quality score ≥ 20. The genomes were *de novo* assembled with a SPAdes assembler v3.14.1 [15] and improved using a Pilon v1.23 [16]. Gene prediction and annotation were performed with Prokka v1.14.5 [17].

ARG prediction was conducted using the Comprehensive Antibiotic Resistance Database (CARD) [18]. The Resistance Gene Identifier (RGI) tool was used for predicting the resistome based on homology and single nucleotide polymorphism (SNP) models with the default parameters. The in silico detection and plasmid typing based on replicon sequence analysis was performed using the PlasmidFinder web tool [19]. The virulome mining was conducted with ABRicate software package against the *E. coli* virulence database based on Virulence Factor Database (VFDB) (https://github.com/tseemann/abricate accessed on 30 July 2021) [20]. 

### 2.3. Phylogenomic Analysis

A global-scale phylogenomic reconstruction was performed with the 376 ST38 *E. coli* genomes. These genomes were annotated using Prokka v1.14.6 [17] and the resultant gff3 files were submitted to Roary v3.13.0] [21] to determine the core genome. The SNP sites of the concatenated core genes (104,645 bp) were extracted using snp-sites v2.5.1 [22] and submitted to IQTree v1.6.12 [23] to obtain a maximum likelihood SNP tree, which used the model of substitution GTR + F + ASC + R8 and 1000 ultrafast bootstrap replicates. The SNP tree was generated using iTOL v4 [24].

## 3. Results and Discussion

### 3.1. Global Epidemiology of ST38

**Figure 1:**
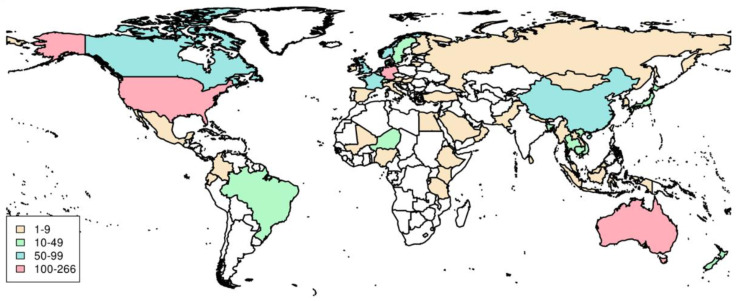
Map of epidemiological distribution of *E. coli* ST38 based on available metadata from EnteroBase.

In the context of a study conducted in a clinical setting in the Amazon region, we identified an ExPEC strain, which was recovered from sputum, belonging to ST38, and carrying the bla_CTX-M-15_ and bla_NDM-1_ resistance genes. Manges et al. [5], based on a systematic revision, had established the worldwide distribution of ExPEC lineages, in which ST38 was in the fourth position on the top 20 ExPEC STs reported in the world. In this revision, ST38 occurrence was lower in the Americas relative to other continents and absent in Africa; not being reported prior to 2000 in the world. Based on this scenario, our finding could represent the index case of this lineage emergence in Brazil. In order to raise the epidemiological scenario of this ExPEC ST, we searched for ST38 information in another database, the EnteroBase, which includes published and unpublished MLST data and geographical information (Table S1) (last updated in November 2022). This meta-analysis revealed that ST38 occurred in 72 countries from all continents, with higher prevalence in the USA, Australia, Germany, and the Netherlands, contrasting with the inferences previously based on reports [5]. Moreover, this ExPEC lineage occurred in at least nine African countries (Figure 1). Even though most of ST38 deposited in EnteroBase was from 2000 onwards, there are strains isolated since 1984 (Table S1). In this way, we corroborated the importance of ST38 as an ExPEC lineage worldwide.

### 3.2. ST38 Phylogenomics

**Figure 2:**
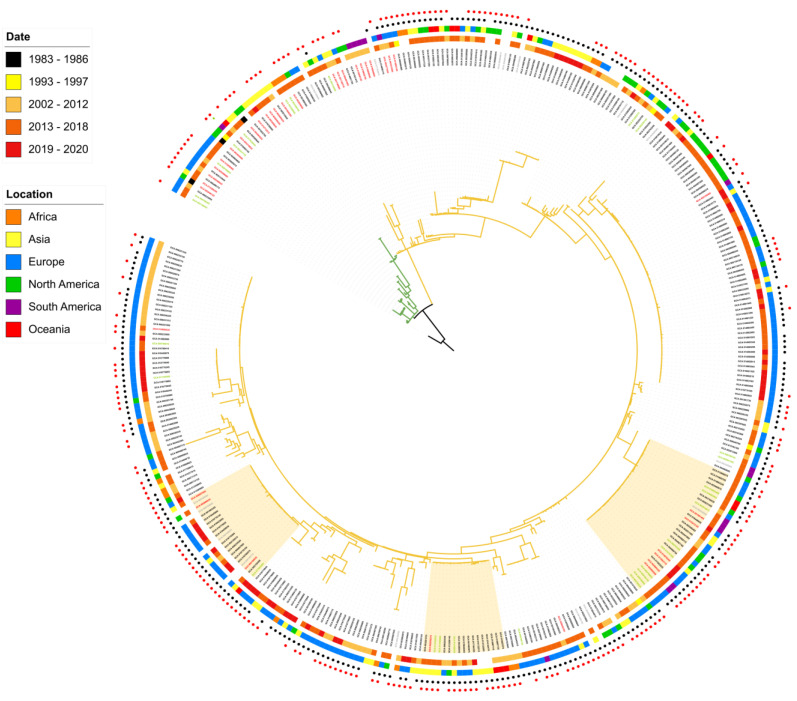
Core SNP tree based on *E. coli* ST38 genomes. Highlighted branches represent Clade 1 (green) and Clade 2 (yellow). The colored circles outside the tree, from innermost to outermost, represent metadata of isolation date and origin, respectively. Genomes carrying the high-pathogenicity island (HPI) and the second T3SS (ETT2) are marked with black and red circles, respectively. The EC35 genome from Brazil obtained in this study is featured by a green star. Highlighted labels represent genomes from human (black), animal (red), environmental (green), and unknown (grey) sources. The yellow hatched regions highlight the occurrence of identical genomes among different sources, demonstrating the zoonotic character of ST38. This figure with a better resolution was also provided in the Supplementary Material (Figure S3).

To gain insights on the ST38 global epidemiology at genomic level, we considered 376 ST38 genomes, retrieved from GenBank and EMBL databases, recovered worldwide from humans, animals, and environmental sources. Interestingly, it was observed that ST38 occurs, at least, for more than three decades (1983–2020), being the oldest strains recovered from animals in the USA (Figure 2). The phylogenomic reconstruction revealed that ST38 genomes grouped in two clades including genomes from all sources (Figure 2). Clade 1 included 32 genomes isolated from 1983 to 2019, while Clade 2 presented several sub-clades containing the majority of genomes (*n* = 344) that were recovered from 2000 to 2020 (Figure 2). It was observed that animal and environmental genomes clustered together with human genomes; however, there are sub-clades represented only by genomes from human sources (Figure 2). Interestingly, identical genomes were observed recovered from humans, animals, and environmental sources in distinct spatio-temporal contexts, stressing the ability and plasticity of some lineages in disseminating among different backgrounds (Figure 2). For instance, there was a strain persisting for more than 30 years in distinct sources in the world: the genome of the Brazilian EC35 strain (GCA_019492145) obtained in 2016, which belonged to a sub-clade in Clade 1, was identical to genomes recovered from poultry in Australia (2008), and from animal and environmental sources in the USA (1986 and 1993, respectively). In the same way, there was a sub-clade in Clade 2 including 31 closely related genomes recovered worldwide from distinct sources between 2012 and 2019. The presence of closely related ST38 genomes globally distributed points to their epidemiological success in terms of persistence and niche adaptability. Recently, an ST38 genomic analysis revealed the presence of a clade comprising genomes from humans, wild and companion animals, and animal feed recovered in Asia, Europe, the Americas, and Australia between 2013 and 2019, stressing the spreading capacity and ubiquity of some ST38 clones [3]. Therefore, these findings altogether demonstrated that ST38 corresponds to a successful zoonotic or zooanthroponosis *E. coli* lineage.

### 3.3. ST38 Acquired Resistome

The analysis of the acquired resistome revealed the presence of a rich and diverse arsenal of antibiotic resistance genes (ARGs) with a quite heterogeneous distribution among ST38 genomes, independently of their source, genetic relatedness, and spatio-temporal context. Overall, genes involved with resistance to most of the clinically relevant antibiotics for treating *E. coli* infections, such as aminoglycosides, β-lactams, including fifth-generation cephalosporins and carbapenems, and fluoroquinolones were found within these genomes, except for those associated with tigecycline and fosfomycin resistance [25]. Although the most prevalent ARGs among ST38 were aph(3″)-Ib, aph(6″)-Id, mphA, mphE, qacEΔ1, and sul, here we discuss ARGs with clinical impact, such as those coding for ESBLs (bla_CTX-M_ and bla_CMY_), carbapenemases (bla_KPC_ and bla_OXA_), metallo-β-lactamases (MBLs) (bla_NDM_, bla_IMP_, and bla_VIM_), and pentapeptide-repeat proteins (qnr family). 

It was found that bla_CTX-M_ alleles (bla_CTX-M-1,-2,-3,-9,-14,-15,-16,-24,-27,-55_) were dispersed in the great majority of ST38 genomes (273/376 genomes), corroborating other studies demonstrating the occurrence of bla_CTX-M_ in the ST38 lineage (Figure S1) [26,27]. The bla_CTX-M-14_ and bla_CTX-M-27_ were the most prevalent among genomes and, in fact, bla_CTX-M-14_ is one of the most predominant genotypes among Enterobacteriaceae in the world [28]. A strong association was observed of bla_CTX-M-27_ with a particular sub-clade that included ST38 genomes recovered from Vietnam, the USA, France, the Netherlands, Germany, Qatar, Australia, and Brazil (the only genome in this sub-clade from an animal source), since 57/60 genomes from this sub-cluster harbored this ESBL allele. In the case of bla_CTX-M-14_, it was the unique allele found in a sub-clade comprising genomes recovered between 2006 and 2019 from Asia, Europe, and Oceania from humans and wild animal (one genome from Mongolia). Similarly, bla_CTX-M-14_ was also found in a sub-clade characterized by genomes from the Americas, Asia, Europe, and Oceania recovered from all sources between 2012 and 2020. Sporadic genomes (*n* = 4) co-harbored two bla_CTX-M_ alleles: bla_CTX-M-15_ + bla_CTX-M-27_ in a genome from Vietnam; bla_CTX-M-15_ + bla_CTX-M-9_ from China; bla_CTX-M-3_ + bla_CTX-M-27_ and bla_CTX-M-14_ + bla_CTX-M-15_ from France. In general, clonal genomes harbored the same bla_CTX-M_ allele. However, eventually, distinct bla_CTX-M_ alleles were found among closely related genomes (GCA_016860125-bla_CTX-M-14_; GCA_904863305-bla_CTX-M-15_; GCA_002442325-bla_CTX-M-27_), indicating events of microevolution or recombination. A diversity of bla_CTX-M_ alleles, mainly bla_CTX-M-14_ and bla_CTX-M-15_, was identified among non-human genomes as previously observed elsewhere [28]. In comparison with bla_CTX-M_, the ESBL-coding bla_CMY-2_ gene was less frequent among ST38, although it was present throughout the clades. 

Carbapenems are drugs of choice to treat infections caused by ESBL-producing Enterobacteriaceae, such as CTX-M-producing ExPEC [29] and, therefore, the co-occurrence of ESBLs and MBLs, for example, bla_NDM-1_, among these strains, is of great global concern due to its clinical outcomes. Here, in spite of the clinical origin of most of the ST38 genomes analyzed, we observed a low prevalence of carbapenem resistance genes (bla_OXA-48_: 11%; bla_OXA-244_: 14%; bla_KPC-2_: 0.8%; bla_NDM-1_: 0.5%; bla_IMP-4_: 0.26%; bla_VIM-4_: 0.26%) (Figure S1). The bla_OXA-48_ and bla_OXA-244_ were found more frequently and spread among the carbapenemase genes analyzed, and *E. coli* ST38 has been associated with the spread of OXA-48 by clonal expansion [11,30,31]. In fact, we observed identical ST38 genomes carrying bla_OXA-48_ in both clinical strains from distinct countries (Netherlands, USA, and Germany) and also in animals (birds/Switzerland). On the other hand, our large-scale resistome analysis revealed that bla_OXA-48_ distribution in ST38 was not only due to clonal expansion since this gene was found spread among unrelated genomes recovered from distinct countries in Asia (Qatar, Lebanon, and Turkey) distributed through several sub-clades (Figure S1). Concerning bla_OXA-244_, a point-mutation derivative of bla_OXA-48_, we verified that this allele was spread by clonal expansion of ST38 in Europe, as previously observed [11], and Asia, but also by horizontal transmission, since bla_OXA-244_ was found in clinical unrelated ST38 genomes from Qatar, Colombia, Switzerland, and Germany, and in an environmental genome from USA. 

Among the 376 ST38 genomes MBL genes were quite rare: bla_NDM-1_ (*n* = 2; Brazil and Switzerland, 2016), bla_IMP-4_ (*n* = 1; China, 1983), and bla_VIM-4_ (*n* = 1; France, 2014) (Figure S1). So far, only bla_NDM_ gene had been reported in a few ST38 strains in Asia [32,33,34,35]. In fact, MBL genes are not highly prevalent in any *E. coli* ST when compared with other Enterobacteriaceae family members [36]. Interestingly, all of these MBL-positive genomes (except for the one from Switzerland) co-harbored a bla_CTX-M_ gene, all of them associated with human infection cases: bla_CTX-M-14_ + bla_CTX-M-15_ + bla_VIM-4_ in a genome recovered in France (GCA_009909835); bla_CTX-M-14_ + bla_IMP-4_, in an isolate from China (GCA_002223705); and bla_CTX-M-15_ + bla_NDM-1_ in the Brazilian EC35 genome obtained in this study (GCA_019492145) (Figure S1). Although rare, such association has a relevant impact due to its clinical burden. 

In contrast, the in vitro resistome analysis of the EC35 strain, recovered in the Amazon region and sequenced in the present study, had revealed an MDR phenotype, being resistant to most antibiotics tested, including the new drug combinations ceftolozane/tazobactam and ceftazidime/avibactam (Table 1). Its resistome was in agreement with this resistance phenotype since, besides bla_CTX-M-15_ and bla_NDM-1_, it harbored genes conferring resistance to cephalosporins (bla_CMY-2_), trimethoprim (dfrA14), sulfonamides (sul2), tetracycline (tet(A)), streptomycin (strBA) and quinolones (qnrS1 and a novel qnrB allele encoding a protein sharing 85% identity with QnrB1). Interestingly, the co-occurrence of these two qnr-type genes might synergistically contribute to the accumulation of activity, increasing quinolone resistance. Indeed, the observed MIC of ciprofloxacin for EC35 (0.5 mg/L) was slightly higher than that related to qnrB1 and qnrS1 alone (MICs 0.1–0.25 mg/L) [37]. This genome is closely related to two genomes from animals (Australia and USA) and one from the environment (water) USA. The resistome of these non-clinical genomes comprised only bla_TEM-181_, dfrA5 and tet(A) (GCA_003318825/animal/Australia) and sul2 (GCA_002463095/animal/USA), while the environmental one (GCA_002231735) had no antibiotic resistance gene. Considering that these genomes are clonal, this finding strongly suggests that the huge resistome presented in the clinical EC35 genome is associated with an acquired mobilome. In fact, the in silico plasmid searches revealed the presence of different plasmid backbones from several incompatibility groups, such as IncHI2 and IncFIb, and most of the ARGs were associated with these plasmid segments. However, due to the technical limitations imposed by the sequencing methodology applied, it was not possible to completely characterized these elements. 

It is worth mentioning the occurrence of an expressive arsenal of ARGs among several non-human genomes involved with resistance emergence to a broad variety of antibiotics. These genomes were recovered from distinct spatio-temporal contexts and were distributed throughout Clade 1. It was the case of the genomes GCA_014712515 (aac(6″)-Ib9, ant(3″)-IIa, aph(3″)-Ib, aph(3’)-Ia, aph(6)-Id, bla_CMY-59_, bla_OXA-10_, qnrVC4, bla_TEM-181_, cmlA6, dfrA14, floR, qacL, sul2, tet(A)), recovered in China from poultry, 2016; GCA_011742905 (aph(3″)-Ib, aph(6″)-Id, aadA5, the carbapenemase bla_OXA-48_, catI, bla_TEM-181_, dfrA17, qac∆E1, sul1, sul2, tet(D)), recovered in Switzerland from the natural environment, 2019; GCA_002901285 (aaca6-Ib-cr6, bla_CTX-M-15_, bla_OXA-1_, aadA5, dfrA17, qac∆E1, sul1, tet(B)), recovered in Brazil from cabbage, 2016; and GCA_008642395 (aac(3″)-IId, aph(3″)-Ib, aph(6)-Id, bla_CTX-M-14_, bla_TEM-181_, aadA5, dfrA17, qac∆E1, sul1, sul2, tet(D)), recovered in Norway from mollusks, 2014.

### 3.4. ST38 Virulome

The ST38 virulome revealed a robust arsenal of virulence-associated genes related to the successful establishment of both intestinal and extraintestinal infections. The ST38 virulome was characterized by genes involved with crucial roles in host–pathogen interactions and adaptability, competitiveness, and colonization capabilities (aslA, hlyE, motAB, nadAB, malX, artJ, clpV, eaeH, hcp, hofBC, matF, ycfZ, ygdB, b2854/b2972, and the operons aec, ent, ppd, ecp, flg, flh, fli, and ycb) [38,39]. A heterogeneous distribution of these genes was observed among ST38 regardless of source, phylogenetic relationship, and spatio-temporal context. 

In addition to the aforementioned virulence factors, the virulome mining revealed interesting and particular features regarding the T3SS type 2 identified in most of ST38 genomes (Figure 2). The ETT2 secretion system corresponds to T3SS type 2 identified in most of *E. coli* that shares a significant relationship with the T3SS coded by the Salmonella pathogenicity island 1 (SPI-1). Although present in many *E. coli* strains, in most cases the ETT2 had undergone extensive mutational attrition, resulting in a functionless system [40,41]. The complete ETT2 system comprises the three operons eprKJIH-epaSRQPO-eivJICAEGF, involved with apparatus formation, and the eip locus, encoding putative effectors, as well as eilA, eicA, and eaeX, encoding a transcriptional regulator, a chaperone, and an outer membrane invasion/intimin-like protein, respectively [41,42,43]. Here, in order to infer the putative functionality of ETT2 revealed in ST38, we performed a screening on the 376 ST38 genomes considering not only the presence of the genes associated with ETT2 but also their identity at the protein level (Figure S2). This analysis showed a remarkable prevalence of the ETT2 entire system, and the high protein identity (90–100%) shared with EAEC 042 ETT2 (reference of functional ETT2) indicating their putative functionality in most of ST38 genomes. 

So far, only a small set of complete and canonical ETT2 has been reported [41,42,43,44,45,46,47,48,49], which includes the EAEC 042 and the ExPEC ST69 lineage. Concerning the distribution and functionality of ETT2 in the ST38 lineage, a recent study demonstrated its occurrence in ST38 with a high level of conservation relative to the canonical one found in EAEC 042 in only two ST38 strains [43]. 

In the virulome of ST38 we also identified the high-pathogenicity island (HPI) which was first described in pathogenic Yersinia species that encodes the siderophore yersiniabactin [50], and has been considered one of the most relevant virulence factors among ExPEC lineages [39,51,52,53,54]. Besides its pivotal role in iron uptake, the HPI orchestrates various virulence mechanisms and optimizes the overall fitness of ExPEC, such as motility, antibiotic tolerance, and exacerbating inflammatory processes [51,52,53,54].

Interestingly, we observed a strong association and high prevalence of HPI in genomes recovered from humans/clinical setting over space and time distributed throughout Clade 2. Conversely, this island was absent in Clade 1, which is composed mainly of animal and environmental ST38 genomes (Figure 2). In fact, previous studies have revealed that HPI is prevalent in strains associated with nosocomial infections [39,51,52,53,54]. Therefore, our results raise genomic evidence that corroborates the HPI association with successful *E. coli* ST38 establishment in human infections. 

## 4. Conclusions

Here, we presented the large-scale genomic analyses of the high-risk ExPEC ST38, focusing on its resistome and virulome, providing novel insights into its global epidemiology in terms of the three axes of the One Health concept. It was demonstrated that ST38 is a host-broad pathogen since clones were found efficiently persisting in humans, animals, and natural environments, underlining its global dissemination. Due to the high prevalence of HPI in human/clinical genomes, we could conclude that HPI plays a major role in the establishment of ST38 as a successful pathogen in humans.

## Figures and Tables

**Table 1:** EC35 antimicrobial resistance profile.

Antibiotics	EC35 (MIC, mg/L)
Ampicillin	≥256
Ampicillin/sulbactam	≥256
Amoxicillin/clavulanate	≥256
Ticarcillin/clavulanic acid	≥256
Piperacillin/tazobactam	≥256
Cefoxitin	128
Ceftriaxone	128
Cefotaxime	64
Cefuroxime	128
Ceftazidime	≥256
Cefepime	32
Ceftolozane/tazobactam	≥256
Ceftazidime/avibactam	≥256
Aztreonam	16
Imipenem	32
Meropenem	16
Ertapenem	32
Doripenem	2 (I)
Tetracycline	64
Trimethoprim/sulfamethoxazole	>32
Ciprofloxacin	0.5 (I)
Streptomycin	96
Gentamicin	4
Tobramycin	4
Amikacin	8
Chloramphenicol	4
Fosfomycin	16
Tigecycline	0.125
Colistin	1.5

## Data Availability

Not applicable.

## Supplementary Materials

The following supporting information can be downloaded at: https://www.mdpi.com/article/10.3390/microorganisms10122482/s1, Figure S1: Heatmap of the ST38 resistome considering the carbapenemase and ESBL genes.; Figure S2: Heatmap of the ST38 virulome considering the ETT2 T3SS genes.; Figure S3: Core SNP tree based on *E. coli* ST38 genomes.; Table S1: Metadata of *E. coli* ST38 isolates/genomes from Enterobase.
