# Supplementary material for: Global Genomic Epidemiology of Escherichia coli (ExPEC) ST38 Lineage Revealed a Virulome Associated with Human Infections
Source: Microorganisms. 2022 Dec 15;10(12):2482. doi: 10.3390/microorganisms10122482 (PMC9787326; doi:10.3390/microorganisms10122482)
Supplement: Supplementary file 1 [file microorganisms-10-02482-s001.zip › HEATMAP ETT2 (Figure S2).pdf]

Figure S2

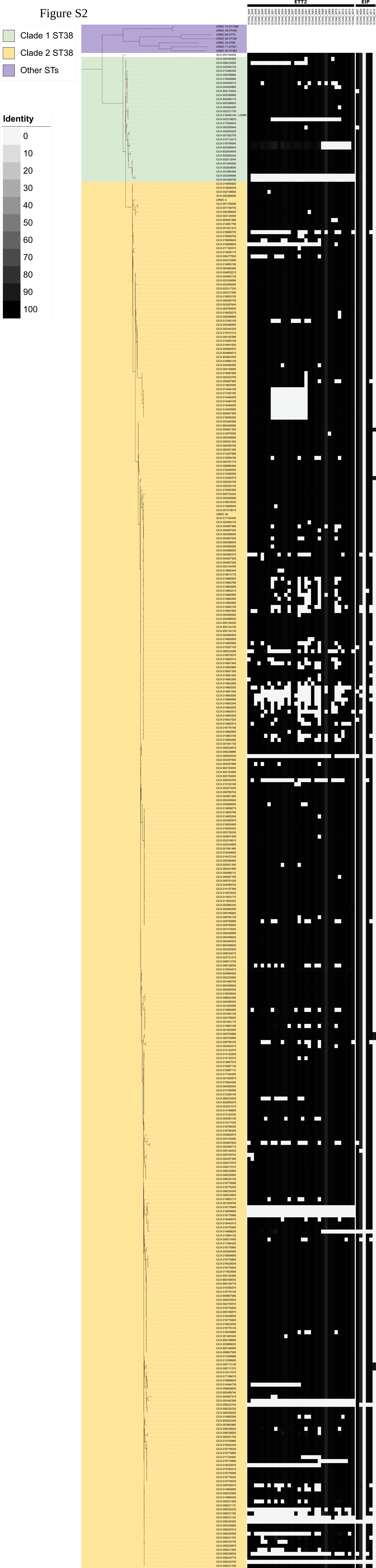
Clade 1 ST38
Clade 2 ST38
Other STs
Identity
0
10
20
30
40
50
60
70
80
90
100
ETT2
EIP
