# Supplementary material for: Global Genomic Epidemiology of Escherichia coli (ExPEC) ST38 Lineage Revealed a Virulome Associated with Human Infections
Source: Microorganisms. 2022 Dec 15;10(12):2482. doi: 10.3390/microorganisms10122482 (PMC9787326; doi:10.3390/microorganisms10122482)
Supplement: Supplementary file 1 [file microorganisms-10-02482-s001.zip › supplementary material legends.pdf]

**Figure S1:** Heatmap of the ST38 resistome considering the carbapenemase and ESBL genes. The core SNP tree is presented with Clade 1 and Clade 2 branches highlighted in green and yellow, respectively. Highlighted labels represent genomes from human (black), animal (red), environmental (green), and unknown (grey) sources. The yellow hatched regions highlight the occurrence of identical genomes among different sources, demonstrating the zoonotic character of ST38. The circles outside the tree correspond to the presence/absence of ARGs in each genome, where colored blocks represent the presence of a specific gene. Each ARG is labeled by a color as shown in the figure legend.

**Figure S2:** Heatmap of the ST38 virulome considering the ETT2 T3SS genes. A phylogenetic tree is presented with Clade 1 and Clade highlighted in green and yellow, respectively, and other *E. coli* STs were highlighted in purple. Red dots indicate bootstrap values >80. The complete and canonical set of genes that comprise the functional secretion system (ETT2 locus) and its corresponding effector proteins (EIP locus) from the EC042 strain were checked against all ST38 genomes. The black and white blocks correspond to the presence and absence, respectively, of each ETT2 and EIP loci gene.

**Figure S3:** Core SNP tree based on E. coli ST38 genomes. Highlighted branches represent Clade 1 (green) and Clade 2 (yellow). The colored circles outside the tree, from innermost to outermost, represent metadata of isolation date and origin, respectively. Genomes carrying the high-pathogenicity island (HPI) and the second T3SS (ETT2) are marked with black and red circles, respectively. The EC35 genome from Brazil obtained in this study is featured by a green star. Highlighted labels represent genomes from human (black), animal (red), environmental (green), and unknown (grey) sources. The yellow hatched regions highlight the occurrence of identical genomes among dif-ferent sources, demonstrating the zoonotic character of ST38.

**Table S1:** Metadata of *E. coli* ST38 isolates/genomes from Enterobase.
