# Supplementary figures and images for: Global Genomic Epidemiology of Escherichia coli (ExPEC) ST38 Lineage Revealed a Virulome Associated with Human Infections
Source: Microorganisms. 2022 Dec 15;10(12):2482. doi: 10.3390/microorganisms10122482 (PMC9787326; doi:10.3390/microorganisms10122482)

Figure S1

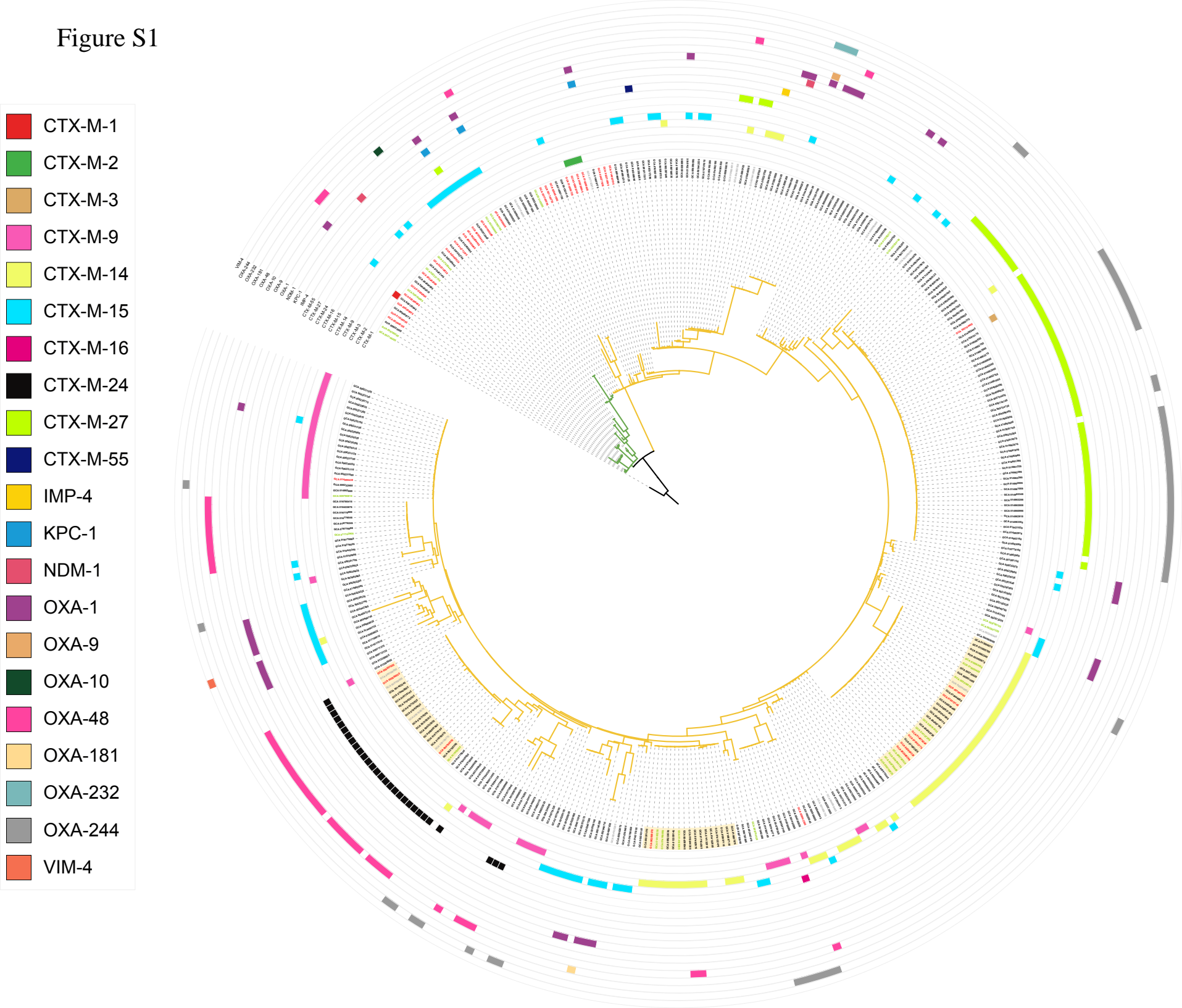

Supplement: Supplementary file 1 [file microorganisms-10-02482-s001.zip › HEATMAP RESISTOMA COLORIDO E CIRCULAR (fIGURE S1).pdf]

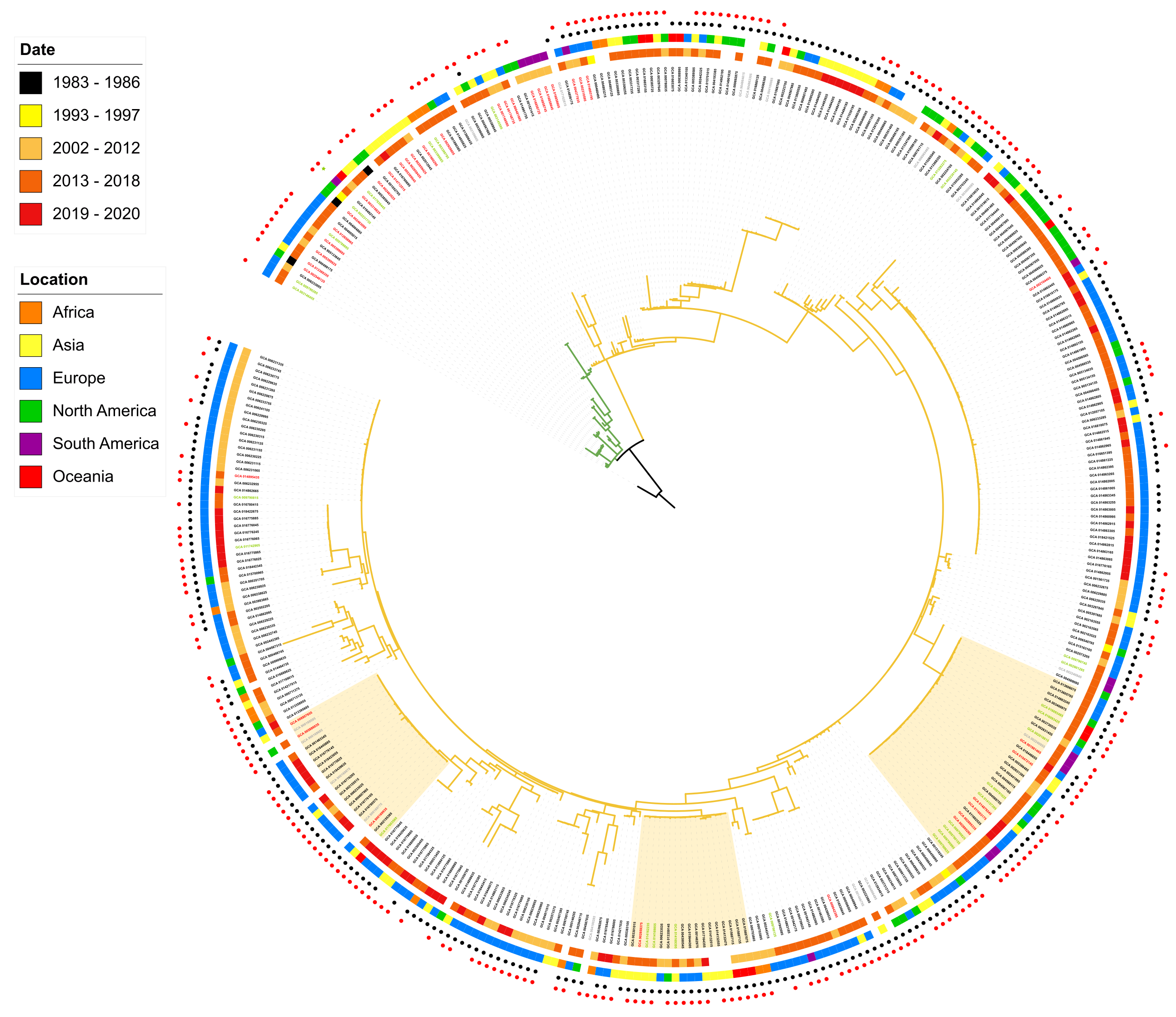

Supplement: Supplementary file 1 [file microorganisms-10-02482-s001.zip › PHYLOGENETIC TREE (MICRORGANISMS) Figure S3.png]
